# HDL functionality is dependent on hepatocyte stress defense factors Nrf1 and Nrf2

**DOI:** 10.3389/fphys.2023.1212785

**Published:** 2023-07-12

**Authors:** Michael J. Trites, Brynne M. Stebbings, Hiroyuki Aoki, Sadhna Phanse, May G. Akl, Lei Li, Mohan Babu, Scott B. Widenmaier

**Affiliations:** ^1^ Department of Anatomy, Physiology and Pharmacology, University of Saskatchewan, Saskatoon, SK, Canada; ^2^ Department of Biochemistry, University of Regina, Regina, SK, Canada; ^3^ Department of Physiology, Faculty of Medicine, University of Alexandria, Alexandria, Egypt

**Keywords:** HDL, liver stress defense, cholesterol, transcription factor, immunometabolism

## Abstract

High density lipoproteins (HDL) promote homeostasis and counteract stressful tissue damage that underlie cardiovascular and other diseases by mediating reverse cholesterol transport, reducing inflammation, and abrogating oxidative damage. However, metabolically stressful conditions associated with atherosclerosis can impair these effects. Hepatocytes play a major role in the genesis and maturation of circulating HDL, and liver stress elicits marked regulatory changes to circulating HDL abundance and composition, which affect its functionality. The mechanisms linking liver stress to HDL function are incompletely understood. In this study, we sought to determine whether stress defending transcription factors nuclear factor erythroid 2 related factor-1 (Nrf1) and −2 (Nrf2) promote hepatocyte production of functional HDL. Using genetically engineered mice briefly fed a mild metabolically stressful diet, we investigated the effect of hepatocyte-specific deletion of Nrf1, Nrf2, or both on circulating HDL cholesterol, protein composition, and function. Combined deletion, but not single gene deletion, reduced HDL cholesterol and apolipoprotein A1 levels as well as the capacity of HDL to accept cholesterol undergoing efflux from cultured macrophages and to counteract tumor necrosis factor α-induced inflammatory effect on cultured endothelial cells. This coincided with substantial alteration to the HDL proteome, which correlated with liver gene expression profiles of corresponding proteins. Thus, our findings show complementary actions by hepatocyte Nrf1 and Nrf2 play a role in shaping HDL abundance and composition to promote production of functionally viable HDL. Consequently, our study illuminates the possibility that enhancing stress defense programming in the liver may improve atheroprotective and perhaps other health promoting actions of HDL.

## Introduction

Circulating high-density lipoprotein (HDL) play several important roles in cardiovascular health, immunometabolism, and homeostasis. This includes mediating reverse cholesterol transport, modulating inflammation as well as coagulation, and abrogating oxidative damage (Gordon et al., 2011; Navab et al., 2011; Fisher et al., 2012; Riwanto and Landmesser, 2013). Moreover, several studies (Gordon et al., 1977; Assmann et al., 1996; The Emerging Risk Factors Collaboration et al., 2012) show there is an inverse correlation between circulating HDL-cholesterol (HDLc) and coronary artery disease (CAD) and that increasing HDL function promotes atheroprotection in humans and preclinical CAD models (Rubin et al., 1991; Degoma and Rader, 2011; Fisher et al., 2012; Feig et al., 2014). However, while enhancing HDL function has been recognized as a potential therapeutic target for CAD, clinical trials designed to enhance HDL function by raising HDLc via inhibiting cholesterol ester transfer protein did not yield the targeted clinical benefit (Tall and Rader, 2018), which raises concern for the utility of HDL targeting therapy.

One explanation for the limited successes with HDL targeting therapy is that circulating HDL may become dysfunctional in the disease context. For example, in atherosclerosis HDL appears prone to acquire dysfunctional and proinflammatory properties that may promote, rather than alleviate, the progression to atherothrombosis (Navab et al., 2011; Rosenson et al., 2016). Indeed, alterations in HDL composition in patients with CAD has been shown to impair the ability of circulating HDL to accept cholesterol from cholesterol-loaded macrophage foam cells, which reduces clearance of cholesterol from atherosclerotic plaques through the reverse cholesterol transport pathway (Rohatgi et al., 2014; Annema et al., 2016). Additional manifestations of dysfunctional HDL include a diminished capacity to exert anti-inflammatory actions (Dullaart et al., 2014; Annema et al., 2016) as well as oxidative damage to the HDL particle (DiDonato et al., 2013; Huang et al., 2014) that coincide with reduced lipid transport proteins and increased proinflammatory proteins (Rosenson et al., 2016). Hence, the qualitative and quantitative features that underlie HDL functionality may be critical determinants for atherosclerosis outcomes. Delineating the mechanisms by which HDL function is established and regulated may reveal critical atherogenic factors or, in contrast, effective approaches to boost the atheroprotective functions of HDL in patients with high CAD risk.

HDL biogenesis is a complex process. In healthy subjects, circulating HDL is ∼50% mass weight of protein that stabilize lipid emulsion containing ∼25% phospholipid, 4% cholesterol, 3% triglyceride, and 12% cholesterol ester (Shah et al., 2013). HDL is initially synthesized in liver or intestine, with approximately 70% of circulating HDL derived from hepatocytes (Timmins et al., 2005; Brunham et al., 2006). The nascent HDL particle is a lipid poor complex that undergoes remodelling in the circulation, which involves acquiring cholesterol, changing size, altering density, and exchanging and modifying resident proteins (Segrest et al., 2000; Kypreos and Zannis, 2007; Gordon et al., 2011; Shah et al., 2013; Zannis et al., 2015). Diverse functions of mature HDL are rendered by the more than 150, and likely much more, proteins that reside on at least one of many circulating subspecies, with apolipoprotein A1 (ApoA1) being most abundant and pervasive (Gordon et al., 2011; Shah et al., 2013; Furtado et al., 2018; Davidson et al., 2022).

HDL protein composition has been observed to undergo dramatic remodelling in subjects exposed to agents that promote organ dysfunction and inflammation, such as metabolic stress, toxins, and pathogens (Carpentier and Scruel, 2002; Khovidhunkit et al., 2004; Haas and Mooradian, 2010; Laudanski, 2021; Webb, 2021). While acute and transient alteration of HDL abundance and composition can contribute to a physiological defense response in specific scenarios, when this effect is unresolved and chronic it may promote functional impairments in HDL that contribute to atherosclerosis progression (Van et al., 1995; McGillicuddy et al., 2009; Gordon et al., 2011; Navab et al., 2011; Rosenson et al., 2016). Identification of defense systems that underlie the orchestration of HDL and its protein composition may uncover insight needed to deconvolute the relationship between stress and HDL function in health and disease.

Hepatocytes produce and secrete the majority of several types of HDL resident protein (Shah et al., 2013; Furtado et al., 2018), including those contributing to lipid transport (e.g., ApoA1, ApoA2, and ApoA4), inflammation (e.g., complement C3 and C9 and ApoJ), as well as coagulation and metal transport (e.g., fibrinogen beta chain and haptoglobin), and altering liver production rate of HDL resident protein has been shown to alter HDL functionality (Zhang et al., 2003). Conversely, exposing the liver to inflammation or injury stimuli can trigger the acute phase response, a hepatocyte-driven stress defense program that results in reduced liver production of ApoA1 and related lipid transport proteins as well as increased production of ApoJ and related inflammatory proteins (Gabay and Kushner, 1999; Carpentier and Scruel, 2002; Khovidhunkit et al., 2004; Rosenson et al., 2016; Webb, 2021). The consequence is altered HDL level, composition, and function. Thus, stress defense networks in hepatocytes seem to play a role in regulating HDL function. However, which stress defenses underlie this process is not understood.

Nuclear factor erythroid 2 related like-1 (Nrf1) and -2 (Nrf2) are homologous stress defense factors belonging to the cap’n’collar family of basic leucine zipper transcription factors. Both molecules can regulate expression of genes that mediate stress defense and resolution as well as promote homeostasis (Zhang and Xiang, 2016; Yamamoto et al., 2018; Cuadrado et al., 2019; Bartelt and Widenmaier, 2020). Studies in mice show Nrf1 (Xu et al., 2005; Hirotsu et al., 2012; Widenmaier et al., 2017) and Nrf2 (Sugimoto et al., 2010; Okada et al., 2013; Mohs et al., 2021) can protect against impaired cholesterol metabolism, liver stress, and fatty liver disease. Recently, we showed that hepatocyte Nrf1 and Nrf2 complementarily regulate gene expression programming that protects against hepatic cholesterol overload (Akl et al., 2023). While undertaking this study, we also discovered that combined hepatocyte deletion of Nrf1 and Nrf2 in mice fed a diet enriched with high fat, fructose, and cholesterol (HFFC) resulted in abnormal circulating cholesterol level, and systematic expression profiling revealed coinciding alteration in liver expression of genes related to HDL function. Hence, these stress defense factors may contribute to HDL functionality. Here, we investigate the role of hepatocyte Nrf1 and Nrf2 on circulating HDL abundance, composition, and function in mice fed HFFC diet. Our results show complementary actions of these stress defense factors promote liver production of HDL that can effectively mediate cholesterol transport and anti-inflammation.

## Materials and methods

**FIGURE 1:**
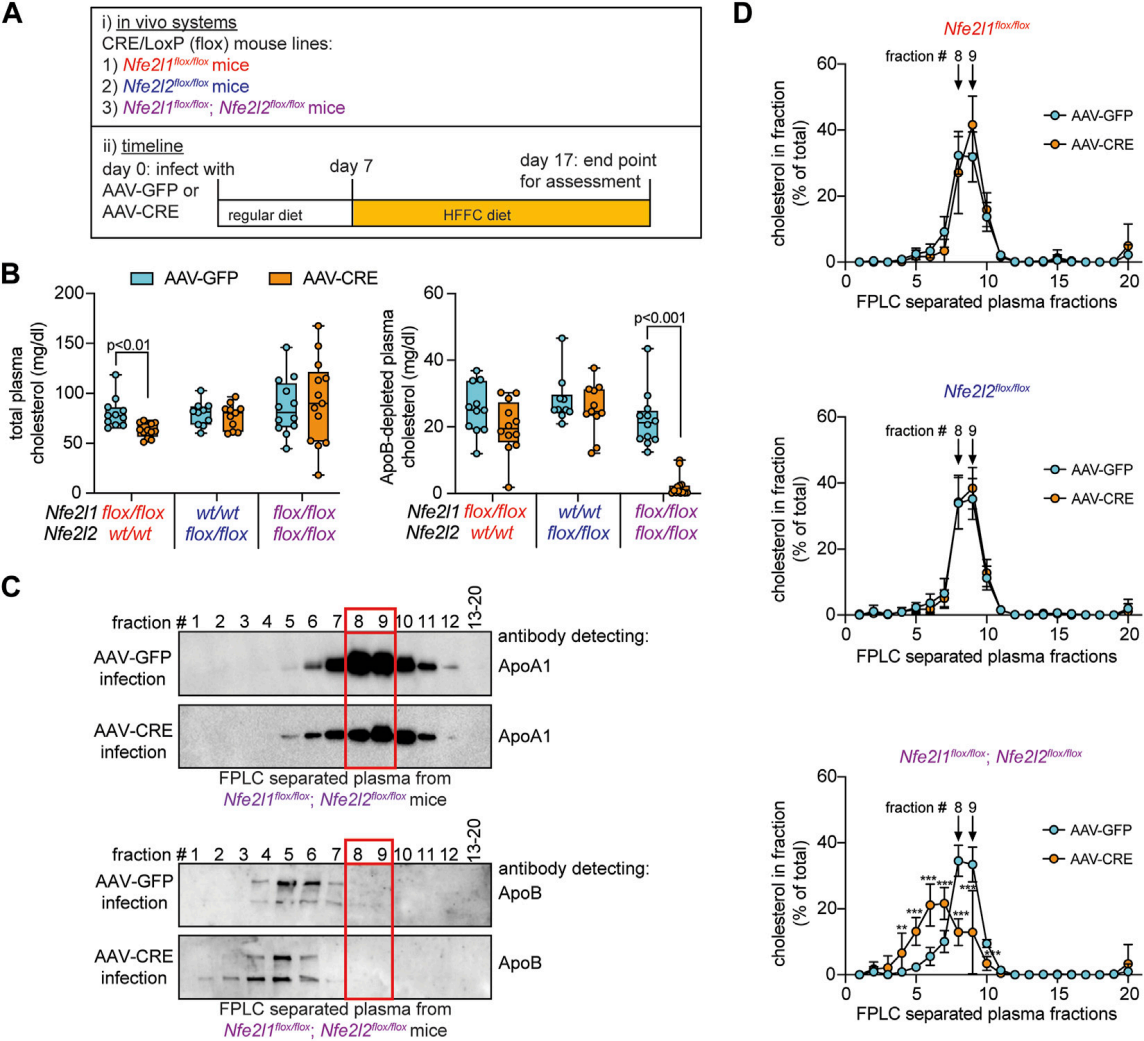
Circulating HDL cholesterol is reduced in mice with combined hepatocyte deficiency of Nrf1 and Nrf2. **(A)** Study design. **(B)** Cholesterol level in plasma and in ApoB-depleted plasma (n = 5-6 males pooled with 5-7 females). **(C)** Representative Western blot of FPLC fractionated plasma showing enrichment of ApoA1 but not ApoB in fractions 8 and 9, identified with a red rectangle in panel. For ApoA1 analysis, samples from two *Nfe2l1* ^*flox/flox*^; *Nfe2l2* ^*flox/flox*^ mice, one *Nfe2l1* ^*flox/flox*^ mouse, and one *Nfe2l2* ^*flox/flox*^ mouse infected with AAV-GFP underwent examination as well as three *Nfe2l1* ^*flox/flox*^; *Nfe2l2* ^*flox/flox*^ mice, two *Nfe2l1* ^*flox/flox*^ mice, and one *Nfe2l2* ^*flox/flox*^ mouse infected with AAV-CRE. For ApoB analysis, samples from three *Nfe2l1* ^*flox/flox*^; *Nfe2l2* ^*flox/flox*^ mice infected with AAV-GFP and three infected with AAV-CRE underwent examination as well as one *Nfe2l2* ^*flox/flox*^ mouse infected with AAV-GFP and one infected with AAV-CRE. **(D)** Cholesterol profiles in FPLC fractionated plasma (n = 5-6 males pooled with 5-6 females). The *p*-value was determined by t-test, adjusted for multiple comparison (**, *p* < 0.01; ***, *p* < 0.001). Data in B is Box and Whisker plot, with points showing the value of each biological replicate. Data in D is mean ± standard deviation.

**Animals used in study**. Animal experiments were done with approval of the University of Saskatchewan’s Animal Care Committee. Experiments were initiated on 8–10 week old male and female C57bl/6 mice. Mice were group housed at 21°C on a 12 h light/dark cycle and provided *ad libitum* access to food and water. Mice were fed regular chow (Prolab RMH 3000; catalog# 5058) from LabDiet or high fat, fructose, and cholesterol (HFFC) diet from Research diets (catalog# D19021910), as indicated in Figure 1A. Mice contained flox alleles in genes for Nrf1 (*Nfe2l1* ^*flox/flox*^), Nrf2 (*Nfe2l2* ^*flox/flox*^), or both (*Nfe2l1* ^*flox/flox*^; *Nfe2l2* ^*flox/flox*^), as described previously (Widenmaier et al., 2017; Akl et al., 2023). To delete Nrf1, Nrf2, or both in hepatocytes, recombination of flox alleles to remove respective gene elements was induced by retroorbital infection of mice, while under isoflurane anesthesia, with 2.0 × 10^11^ particles of a liver targeting serotype 8 adeno-associated virus expressing Cre recombinase via hepatocyte-specific thyroxine binding globulin promoter (AAV-CRE), as in previous (Akl et al., 2023). Littermate controls received virus expressing green fluorescent protein (AAV-GFP). AAV-CRE (AAV8.TBG.PI.Cre.rBG) and AAV-GFP (AAV8. TBG.PI.eGFP.WPRE.bGH) were acquired from the University of Pennsylvania Vector Biocore.

**Tissue collection.** Tissues were collected from mice that were fasted for 3 hours, anesthetized with 3% isoflurane at an oxygen flow rate of 1 L/min, and euthanized by cervical dislocation. Blood was collected into a 26 gauge 1 ml syringe needle via intracardiac puncture and placed into a tube containing ethylenediaminetetraacetic acid (5 mM final concentration). Plasma was separated via 8000 g centrifugation at 4°C for 10 min. One aliquot of plasma was combined with sucrose buffer (final concentration: 10% w/v sucrose, 48 μM EDTA, 30 mM NaCl) in preparation for separation by fast-protein liquid chromatography (FPLC). Additional plasma aliquots were placed into a fresh tube and snap frozen on dry ice. To collect liver, vasculature of anesthetized mice was flushed using calcium and magnesium free phosphate buffered saline (PBS). Liver was excised and immediately frozen on dry ice. All tissues were stored at −80°C until further use.

**Fractionation of plasma by fast protein liquid chromatography (FPLC).** Plasma fractionation was done at the University of Alberta Faculty of Medicine and Dentistry’s Lipidomics Core (RRID:SCR_019176). Briefly, 100 μl of EDTA-treated plasma in sucrose buffer were injected by autosampler into an Agilent 1200 high performance liquid chromatography instrument equipped with a Superose 6 Increase 10/300 gel-filtration FPLC column (Cytiva Life Sciences), which separates intact lipoproteins by size. Separation was performed isocratically at 500 μl/min using 150 mM NaCl with 3 mM NaN3 as the mobile phase. Effluent was monitored in real-time at 280 nm and the data analyzed using Agilent Chemstation software. 2-min fractions of 1 ml each were collected, and frozen at −20°C immediately after run completion.

**Analysis of cholesterol.** Cholesterol levels in plasma and plasma fractions were determined using an Amplex Red Cholesterol Assay Kit (Invitrogen, Burlington, Ontario, Canada, A12216) according to the manufacturer’s instructions.

**Proteomics.** FPLC separated fractions 8 and 9 were submitted for proteomic analysis at Dr. Mohan Babu’s laboratory. Protein-containing sample was used for comparative proteomic analysis. To carry out trypsin digestion, supernatant was diluted with 50 mM ammonium bicarbonate pH 7.8, 1 mM 1,4-dithiothreital, 0.05% ProteaseMax Surfactant, and then incubated for 30 min at 37°C with 2 μg trypsin from Pierce (MS-Grade). Then, 2-chloroacetamide was added to a concentration of 0.5 mM. After 8 h, trifluoroacetic acid was added to stop digestion. Digested samples were desalted with Top-Tip C-18 cartridge. Peptide samples were loaded on the column and centrifuged at 500 g for 5 min. After washing by water with 0.1% formic acid solution, peptide samples were eluted by 100 μl of elution solution (60% acetonitrile, 0.1% formic acid in water).

Samples were analyzed by nanoLC coupled to Orbitrap Elite mass spectrometer (Thermo Fisher Scientific). Chromatographic separation of peptides was performed on a Proxeon EASY nLC 1000 System equipped with a Thermo Scientific™ Acclaim™ PepMap™ C18 column, 15 cm × 50 μm ID, 3 μm, 100 Å employing a water/acetonitrile/0.1% formic acid gradient. 5 μl of the samples were loaded onto the column for 100 min at a flow rate of 0.30 μl/min. Peptides were separated using 1% acetonitrile and increasing to 3% acetonitrile in the first 2 min and then using a linear gradient from 3% to 24% of acetonitrile for 170 min, followed by gradient from 24% to 100% of acetonitrile for 29 min and wash 10 min at 100% of acetonitrile. Eluted peptides were directly sprayed into mass spectrometer using positive electrospray ionization (ESI) at an ion source temperature of 250°C and ionspray voltage of 2.1 kV. Full-scan MS spectra (m/z 350–2000) were acquired in the Orbitrap elite at 60 000 (m/z 400) resolution. The automatic gain control settings were 1e6 for full FTMS scans and 5e4 for MS/MS scans. Fragmentation was performed with collision-induced dissociation (CID) in the linear ion trap when ions intensity was >1500 counts. The 15 most intense ions were isolated for ion trap CID with charge states ≥2 and sequentially isolated for fragmentation using the normalized collision energy set at 35%, activation Q at 0.250 and an activation time of 10 M s. Ions selected for MS/MS were dynamically excluded for 30 s. Calibration was performed externally with Pierce LTQ Velos ESI Positive Ion Calibration Solution (ThermoFisher Scientific, catalog #88322). The Orbitrap Elite mass spectrometer was operated with Thermo XCalibur software. All RAW files were converted to mzXML using ReAdW-4.3.1.

Proteomic analysis was in accordance with established methods by Cox and Mann (Cox and Mann, 2008). Raw files were searched in MaxQuant Version 1.6.7.0 against the *Mus musculus* canonical Swiss-Prot proteome downloaded from UniProt on July 24, 2020. Two missed cleavage events were allowed and carbamidomethylation of cysteine was set as a fixed modification while variable modifications were oxidation of methionine and acetylation of protein N-termini. Peptide search tolerance was set to 4.5 parts per million for MS1, and MS2 fragment tolerance was set to 20 parts per million. Both false discovery rates at peptide-spectrum match and protein levels were set to 0.01 and peptides with minimum 7 amino acids were considered for identification.

**RNA extraction, cDNA synthesis, and qPCR.** RNA was extracted from tissue samples and cultured cells using TRIzol according to the manufacturer’s protocol. RNA clean-up was performed on a Qiagen RNeasy plus column (catalog# 74034). Concentration and quality of isolated RNA was determined using a NanoDrop One spectrophotometer (Thermo-Fisher Scientific). One μg of RNA was reverse transcribed into cDNA using a Maxima First Strand cDNA synthesis kit, with dsDNase (ThermoFisher, catalog# K1672). Quantitative PCR (qPCR) was performed with a Bio Rad CFX384-well Real-time PCR Detection System using PowerUp SYBR Green Master Mix (ThermoFisher, catalog# A25742). A list of primer sequences for measuring gene expression are provided in Table 1. Changes in expression of target genes were normalized to reference gene *36b4* and data are presented as relative expression compared to the control group.

**TABLE 1:** List of qPCR primers.

Gene name	Primer sequence
36b4	F: AGGGCGACCTGGAAGTCC
	R: CCC​ACA​ATG​AAG​CAT​TTT​GGA
apoa1	F: GGC​ACG​TAT​GGC​AGC​AAG​AT
	R: CCA​AGG​AGG​AGG​ATT​CAA​ACT​G
apoa2	F: GCA​GAC​GGA​CCG​GAT​ATG​C
	R: GCT​GCT​CGT​GTG​TCT​TCT​CA
apoa4	F: CCA​ATG​TGG​TGT​GGG​ATT​ACT​T
	R: AGT​GAC​ATC​CGT​CTT​CTG​AAA​C
apoe	F: CTG​ACA​GGA​TGC​CTA​GCC​G
	R: CGC​AGG​TAA​TCC​CAG​AAG​C
apoj	F: CCT​TGC​TCA​ACA​GTT​TAG​AGG​AA
	R: CAT​CAT​GGT​CTC​GTT​ACA​CAC​TT
c3	F: GAG​CGA​AGA​GAC​CAT​CGT​ACT
	R: TCT​TTA​GGA​AGT​CTT​GCA​CAG​TG
c9	F: GGG​TGT​CAA​TGC​ACA​GAT​GC
	R: GAG​GCA​AGG​ATC​ACA​CTC​TGA
cpn2	F: GAC​ACC​AAC​TAC​GAC​CTC​TTC​A
	R: TGG​TTG​TTG​TAA​AGA​CGG​AGC
fgb	F: ACT​ACG​ATG​AAC​CGA​CGG​ATA
	R: GGC​AGG​TCT​TAG​GCT​AGG​AG
fgg	F: ACC​AGA​GAT​AAC​TGT​TGC​ATC​CT
	R: CCA​CGT​CGG​TTT​GGT​AAG​AAG
gpld1	F: TCG​AGA​GAA​CTA​CCC​TCT​GCC
	R: GGA​ACC​CTT​GTT​CAA​TAC​CCA​G
Hp	F: GCT​ATG​TGG​AGC​ACT​TGG​TTC
	R: CAC​CCA​TTG​CTT​CTC​GTC​GTT
hrg	F: TGC​TCA​CCA​CAG​CAT​TGC​TT
	R: CAC​TCC​TCC​GCC​CTT​TAT​TGA
itih2	F: GCC​ACA​ACT​ACC​ATC​CAG​AGC
	R: TGT​CAT​GCC​GTT​CAC​AGT​CAT
itih4	F: GTG​GAA​CCT​TGT​GCT​GTT​CTT
	R: CTC​GGC​AGT​AGT​GGT​CGG​A
mcp1	F: GAT​CAC​CAG​CAG​CAG​GTG​T
	R: ATG​TAT​GTC​TGG​ACC​CAT​TCC​TT
Tf	F: TGG​GGG​TTG​GGT​GTA​CGA​T
	R: AGC​GTA​GTA​GTA​GGT​CTG​TGG
vcam1	F: AGT​TGG​GGA​TTC​GGT​TGT​TCT
	R: CCC​CTC​ATT​CCT​TAC​CAC​CC

**Western blotting.** Protein levels in total plasma and FPLC separated plasma were determined by Western blotting. To assess total plasma samples, 1 μl of plasma diluted in 1% sodium dodecyl sulfate containing loading buffer was analyzed from each animal, separated in NuPAGE MOPS Running Buffer in a Novex a 4%–12% Bis-Tris Gel (catalog# NP0336), transferred to nitrocellulose membrane, and probed for proteins of interest using primary antibodies, provided in Table 2. Quantitation of relative protein expression was performed using ImageJ (Schneider et al., 2012), with total protein determined by Ponceau S staining to serve as a loading control. To assess FPLC separated plasma samples, the equivalent of 0.25% of fraction in loading buffer volume was loaded into a well. Blots examining ApoA1 expression were conducted as described for total plasma. To determine ApoB, samples were separated in a Novex 7% tris-acetate gels (catalog# EA0355) in NuPAGE Tris-Acetate Running Buffer (catalog# LA0041).

**TABLE 2:** List of antibodies used in western blotting.

Protein name	Supplier	Catalogue #
apoA1	Abcam	ab20453
apoA2	Invitrogen	PA5-114866
apoB	Abcam	ab20737
apoE	Abcam	ab183597
apoJ	Invitrogen	PA5-86452
Hp	Invitrogen	PA5-102426

**Preparation of ApoB-depleted plasma for cell culture-based HDL function assays.** HDL functional assays using ApoB depleted plasma was done similar to clinical studies on human plasma (Dullaart et al., 2014; Rohatgi et al., 2014; Saleheen et al., 2015; Annema et al., 2016). The ApoB depleted plasma was prepared by combining a 30 μl aliquot of plasma with 15 μl of 36% polyethylene glycol (PEG600, Millipore Sigma, Oakville, Ontario, Canada 528877-100 GM) in 10 mM-HEPES (pH = 8.0), followed by a 30-min incubation on ice. After 30 min the mixture was centrifuged at 2200 g and 4°C for 10 min, as has been described for analysis of HDL function in human plasma sample (Annema et al., 2016). The absence of ApoB was confirmed by Western blot analysis comparing ApoB-depleted plasma to total plasma from the same mice. The HDL-containing supernatant was collected, placed in a fresh tube, kept on ice, and used directly within 4 h of collection for HDL function assays.

The ability of HDL to accept cholesterol was determined via cholesterol efflux assay kit (Abcam, catalog# ab196985) according to the manufacturer’s instructions with minor modifications. Briefly, 5 × 10^4^ RAW264.7 cells from American Type Culture Collection (ATCC, catalog# TIB-71) were added to each well of a 96-well plate (Millipore Sigma, catalog# CLS3595) in 100 μl Dulbecco’s Modified Eagle Medium (DMEM) from Gibco (catalog# 11965–092) with 10% fetal calf serum (FCS) from Hyclone (catalog# SH30087.03) in a humidified cell culture incubator (37°C/5% CO_2_). Cells were allowed to adhere for 6 h. After adherence, cells were washed twice with 100 μl serum-free DMEM, incubated with labelling reagent for 60 min in a humidified cell culture incubator, washed once with 100 μl serum-free DMEM, and incubated in equilibrium buffer overnight (16 h). The next morning, cells were washed twice with 100 μl serum-free DMEM and incubated with 5 μl of ApoB depleted plasma, prepared as described above, and then incubated for 4 h to allow HDL uptake of labelled cholesterol that underwent efflux from macrophage. After the incubation, HDL-containing supernatant was transferred to a 96-well black-walled culture plate (ThermoFisher, catalog# 165305). Fluorescence was determined using a Synergy HT microplate reader (Ex/Em: 485/523 nm). Then, cells were lysed and lysate transferred to a 96-well black-walled culture plate and fluorescence was determined. Cholesterol efflux was calculated by dividing the fluorescence of HDL-containing supernatant by the combined fluorescence sum of supernatant and cell lysate fluorescence, then multiplying by 100. Percent efflux was corrected by subtracting the value for negative control, which was samples from which no ApoB-depleted plasma was added.

The ability of HDL to suppress tumor necrosis factor α (TNFα) mediated vascular cell adhesion protein 1 (*Vcam1*) mRNA expression by endothelial cells was analyzed via incubating C166 endothelial cells (ATCC, catalog# CRL-2581) with ApoB-depleted plasma and TNFα. Briefly, 2.5 × 10^5^ C166 cells were added to each well of a 24-well cell culture plate (Sigma, catalog# CLS3524) and incubated overnight in 500 μl DMEM with 10% FCS. The next morning, cells were washed twice with 300 μl serum-free DMEM and 300 μl of serum-free DMEM was added to each well. Then, 15 μl of ApoB depleted plasma was added and cells were incubated for 30 min. After 30 min, cells were treated with 10 ng/mL recombinant human TNFα (Novus Biologicals, catalog# 210-TA-020). After a 6-h incubation cells were washed, lysed in Trizol (Invitrogen, catalog# 15596018), and then stored at −80 °C until RNA extraction.

The ability of HDL to suppress lipopolysaccharide (LPS)-mediated monocyte chemoattractant protein 1 (*Mcp1*) mRNA expression by macrophages was assessed using RAW264.7 cells. Briefly, 5.0 × 10^5^ RAW264.7 cells were added to each well of a 24-well plate and incubated overnight in 500 μl DMEM with 10% FCS. The next morning, cells were washed twice with 300 μl serum-free DMEM and 300 μl serum-free DMEM was added to each well. Then, 15 μl of ApoB depleted plasma was added to cells. After a 60-min incubation cells were treated with 5 ng/mL LPS from *Escherichia coli* O11:B4 (Sigma, catalog# L2630). After 6 h cells were washed, lysed in Trizol, and then stored at −80°C until RNA extraction.

**FIGURE 2:**
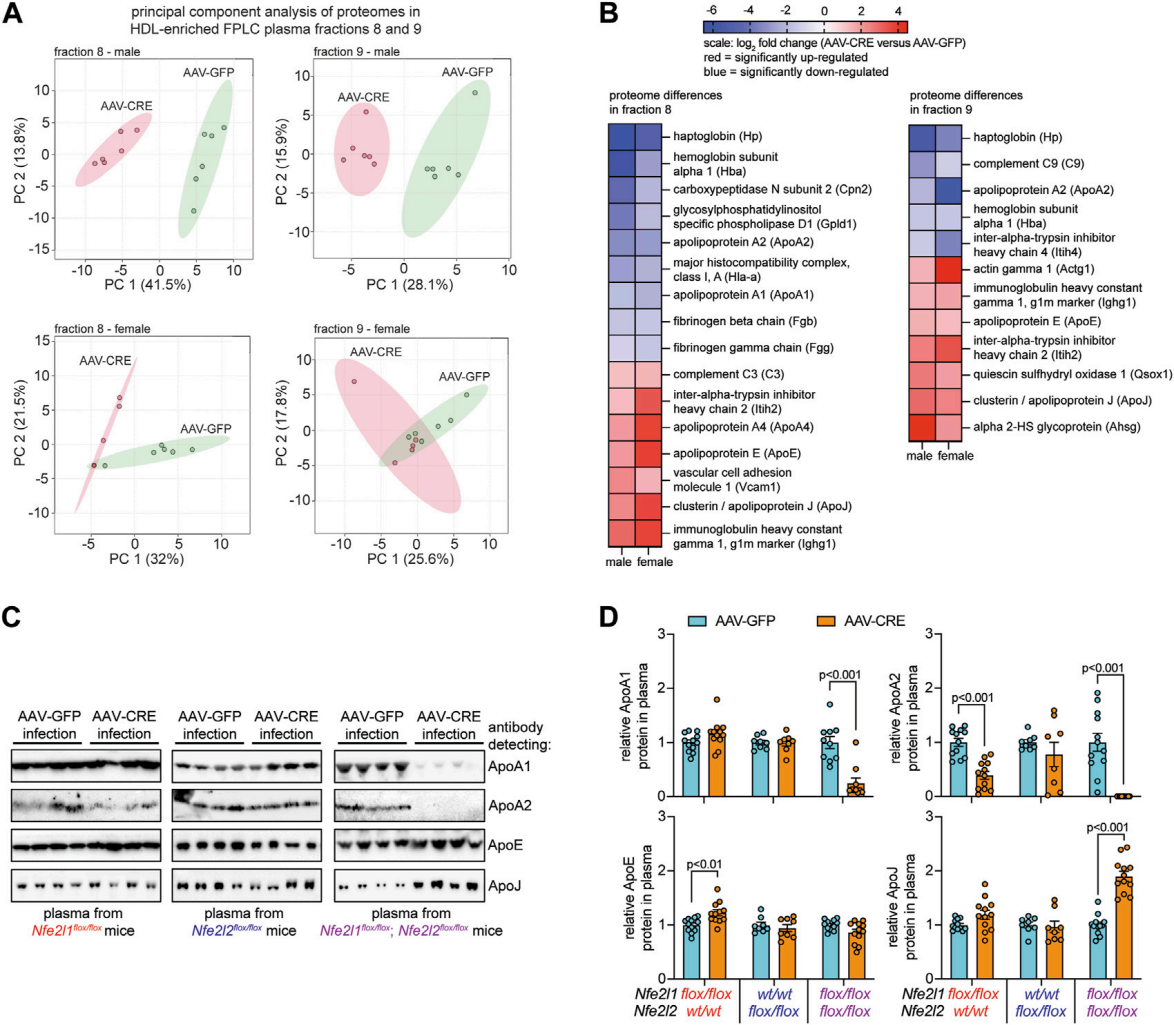
Combined hepatocyte deficiency of Nrf1 and Nrf2 alters the proteome of circulating HDL. **(A, B)** Proteome analysis of FPLC fractions 8 and 9 identified in Figure 1 for males and females (n = 5-6), comparing hepatocyte deficiency of Nrf1 and Nrf2 (AAV-CRE) to control (AAV-GFP). Entails principal component analysis **(A)** and a heat map showing log_2_ fold change differences for the indicated proteins **(B)**. Blue indicates downregulation and red indicates upregulation. **(C, D)** Representative Western blot of indicated proteins in total plasma collected from male mice **(C)** and corresponding analysis **(D)** of these proteins (n = 4-6 males pooled with 4-6 females). The *p*-value was determined by t-test, adjusted for multiple comparison. Data in D is mean ± standard error of the mean, with points showing the value of each biological replicate.

**Statistical Analysis and data availability.** Male and female mice were used throughout and data were pooled for analysis, except for Figures 2A, B. Sample size of males and females was similar or equal, and these details are provided in the figure legends. Except for proteomics, data analysis was done using GraphPad PRISM (version 9.3.1). Significance was defined as *p* < 0.05, using t-test with adjustment for multiple comparisons or regression analysis as indicated in figure legends. Proteomic analysis was done using MetaboAnalyst 4.0 R package (Chong et al., 2018).

## Results

### Hepatocyte Nrf1 and Nrf2 deficiency alters circulating HDL cholesterol level

Previously (Akl et al., 2023), we showed combined hepatocyte Nrf1 and Nrf2 deletion increases liver and circulating cholesterol levels in mice fed high fat, fructose, and cholesterol (HFFC) diet. Here, we used this same system to investigate the role of hepatocyte Nrf1 and Nrf2 on circulating HDL. Hepatocyte Nrf1, Nrf2, or both Nrf1 and Nrf2 were deleted via infecting male and female mice with adeno-associated virus that express CRE recombinase in hepatocytes (AAV-CRE). Littermate control mice were infected with virus expressing green fluorescent protein (AAV-GFP). Seven days after AAV-CRE or AAV-GFP infection, mice were switched to HFFC diet which they were then fed for 10 days (Figure 1A). At time of euthanasia, we collected liver and plasma for analysis. Similar to our previous study (Akl et al., 2023), Nrf1 deficiency resulted in blunted weight gain whereas Nrf2 deficiency had no effect and combined deficiency resulted in weight loss (Supplementary Figure S1A). For all groups, there was no difference in liver weight as a normalized percent of total body weight (Supplementary Figure S1B).

After validating deficiency models, we measured total cholesterol and HDL (i.e., ApoB depleted plasma) cholesterol in plasma (Figure 1B). Compared to respective control, Nrf1 deficiency modestly, but significantly, reduced total cholesterol but had no effect on HDL cholesterol, whereas Nrf2 deficiency had no effect. In contrast, Nrf1 and Nrf2 combined deficiency had no effect on total cholesterol but dramatically reduced HDL cholesterol. Next, plasma samples for each mouse underwent size-based fractionation via fast protein liquid chromatography (FPLC) to separate lipoproteins. To distinguish HDL from non-HDL fractions, Western blots were done to identify which were enriched with the HDL marker, ApoA1, and not with ApoB, a marker of non-HDL lipoproteins. Fractions 8 and 9 were identified as the most robust and consistent (Figure 1C; highlighted in red). Then, we measured cholesterol in each fraction to assess its proportional distribution (Figure 1D). Relative to control, Nrf1 deficiency and Nrf2 deficiency had no effect. In contrast, Nrf1 and Nrf2 combined deficiency reduced the percent of cholesterol in HDL fractions 8–10 while correspondingly increasing the percent of cholesterol in ApoB-rich fractions 4–7. This shift in cholesterol distribution from HDL to ApoB rich lipoprotein may be the reason HDL cholesterol was reduced but total cholesterol unchanged in plasma of mice with combined deficiency (see Figure 1B) and shows complementary actions by hepatocyte Nrf1 and Nrf2 contribute to HDL cholesterol level.

### Hepatocyte Nrf1 and Nrf2 deficiency alters circulating HDL protein composition

Since HDL cholesterol was reduced in mice deficient for hepatocyte Nrf1 and Nrf2 but not in deficiency of either alone, we postulated that this resulted because complementary actions by these factors contribute to HDL protein composition. To test this, proteomic analysis was done on fractions 8 and 9 of plasma from male and female mice with combined hepatocyte Nrf1 and Nrf2 deficiency and compared to AAV-GFP controls. Principal component analysis show proteomes for each fraction cluster distinctly in combined deficiency relative to control (Figure 2A), and we identified several HDL-resident molecules for which protein abundance was significantly different (Figure 2B). This includes proteins involved in lipid metabolism (ApoA1, ApoA2, ApoE), inflammation (ApoJ, C3, C9), and clotting (Fgg, Fgb). To verify these findings and relate it to the effects of single gene deficiency, Western blots were performed on plasma from mice with hepatocyte deficiency for Nrf1, Nrf2, or both and compared to respective controls (Figures 2C, D). For this, we measured a subset of HDL specific lipid transport proteins (i.e., ApoA1, ApoA2), an HDL non-specific protein (i.e., ApoE), and an HDL specific protein involved in the acute phase response (i.e., ApoJ). Nrf1 deficiency had no effect on ApoA1 and ApoJ, reduced ApoA2, and modestly increased ApoE, whereas Nrf2 deficiency had no effect on these proteins. In contrast, combined deficiency substantially reduced ApoA1 and ApoA2, increased ApoJ, and had no effect on ApoE, which can reside on ApoB containing lipoproteins observed in fractions 4–7 (Figure 1C). These results show complementary actions by hepatocyte Nrf1 and Nrf2 contribute to HDL protein composition.

### Hepatocyte Nrf1 and Nrf2 deficiency alters liver expression of HDL resident proteins

Hepatocytes are a primary source of several HDL resident proteins and can alter HDL abundance and composition upon exposure to stressful stimuli (Gabay and Kushner, 1999; Carpentier and Scruel, 2002; Shah et al., 2013; Zannis et al., 2015; Furtado et al., 2018; Laudanski, 2021; Webb, 2021). We reasoned Nrf1 and Nrf2 in hepatocytes may contribute to HDL functionality by promoting liver production of HDL resident proteins. Accordingly, we predicted combined deficiency will result in altered liver expression of HDL resident molecules in a manner that correlates with the altered HDL proteome. To investigate, we identified 14 HDL proteins as being altered in male and female mice with combined deficiency, primarily expressed in hepatocytes, and involved in lipid transport, inflammation, or clotting and metal transport. These are ApoA1, ApoA2, ApoA4, ApoE, ApoJ, carboxypeptidase n2 (Cpn2), complement C3 (C3) and C9, fibrinogen beta chain (Fgb) and gamma chain (Fgg), glycosylphosphatidylinositol specific phospholipase D1 (Gpld1), haptoglobin (Hp), and inter-alpha-trypsin inhibitor heavy chain 2 (Itih2) and Itih4.

**FIGURE 3:**
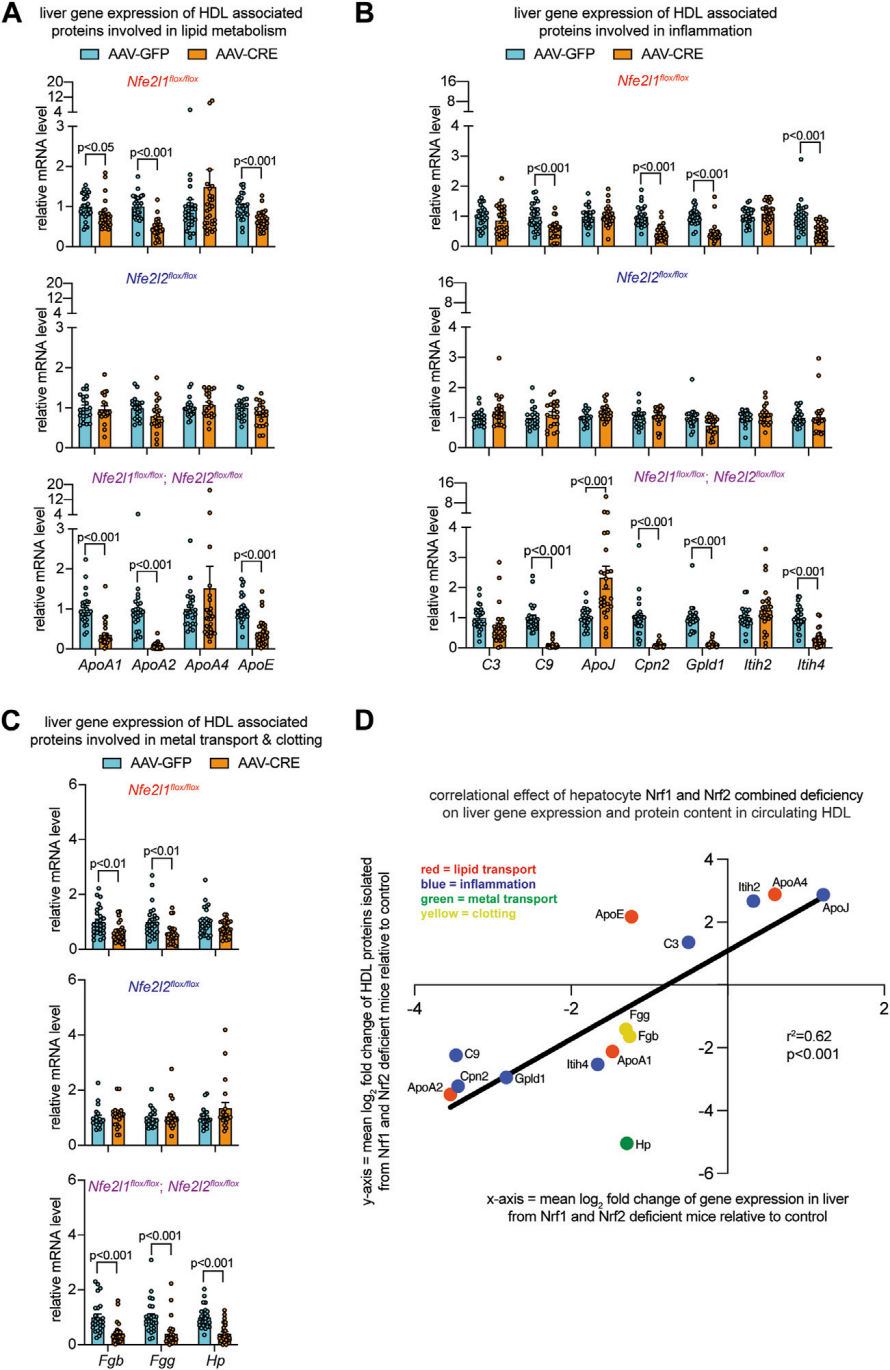
Altered liver gene expression correlates with altered circulating HDL proteome in mice with combined hepatocyte deficiency of Nrf1 and Nrf2. **(A–C)** Liver qPCR analysis for indicated gene expression, normalized by ribosomal protein *36b4* (n = 10-14 males pooled with 10–14 females), to assess liver expression of HDL-resident molecules involved in lipid metabolism **(A)**, inflammation **(B)**, and metal binding and clotting **(C)**. The *p*-value was determined by t-test, adjusted for multiple comparison. Data in **(A–C)** are mean ± standard error of the mean with points showing the value of each biological replicate. **(D)** Correlation of log_2_ fold change in HDL proteome with respect to log_2_ fold change in liver gene expression, comparing combined hepatocyte deficiency of Nrf1 and Nrf2 to respective control. Correlational effect is indicated by r^2^ value and *p*-value was determined using simple linear regression analysis. Each molecule is indicated, and its biological relationship color coded as shown in panel.

To evaluate liver gene expression, quantitative polymerase chain reaction was done. We compared Nrf1 deficiency, Nrf2 deficiency, and Nrf1 and Nrf2 combined deficiency to the respective controls (Figures 3A–C). Nrf2 deficiency had no effect. In contrast, Nrf1 deficiency and combined deficiency altered expression of several genes in a similar manner. These effects were reduced mRNA expression of lipid transport molecules *ApoA1*, *ApoA2*, and *ApoE* (Figure 3A), inflammation molecules *C9*, *Cpn2*, *Gpld1*, and *Itih4* (Figure 3B), and clotting molecules *Fgb* and *Fgg* (Figure 3C). In combined deficiency, there was also reduced expression of metal transport molecule *Hp* (Figure 3C) and increased expression of inflammatory molecule *ApoJ* (Figure 3B). Altogether, this suggests Nrf1 deficiency, rather than Nrf2 deficiency, may be predominantly though not exclusively responsible for altering HDL in mice with combined deficiency. To relate altered liver expression and HDL composition in mice with combined deficiency, relative fold change of these proteins in circulating HDL was correlated with relative fold change in liver gene expression, which resulted in significant positive correlation coefficient of 0.62 (Figure 3D). This evidence is supportive of our postulate that complementary gene transcription programming by Nrf1 and Nrf2 in hepatocytes contribute to HDL protein composition.

### Hepatocyte Nrf1 and Nrf2 deficiency impairs HDL functionality

HDL functionality has been accurately determined in clinical blood samples using APOB depleted plasma (Dullaart et al., 2014; Rohatgi et al., 2014; Saleheen et al., 2015; Annema et al., 2016). Although these samples contain other non-HDL molecules that can influence lipid metabolism and inflammation (e.g., lipids and lipid transfer proteins), it has been reported that approximately 85% of the reverse cholesterol transport and anti-inflammatory activity in APOB depleted plasma from human subjects is attributable to HDL (Jia et al., 2021). Here, we investigated whether altered HDL composition in combined deficiency coincided with diminished cholesterol carrying capacity and anti-inflammatory activity, using cell culture-based assays that are similar to those described for the above-mentioned studies on clinical samples.

**FIGURE 4:**
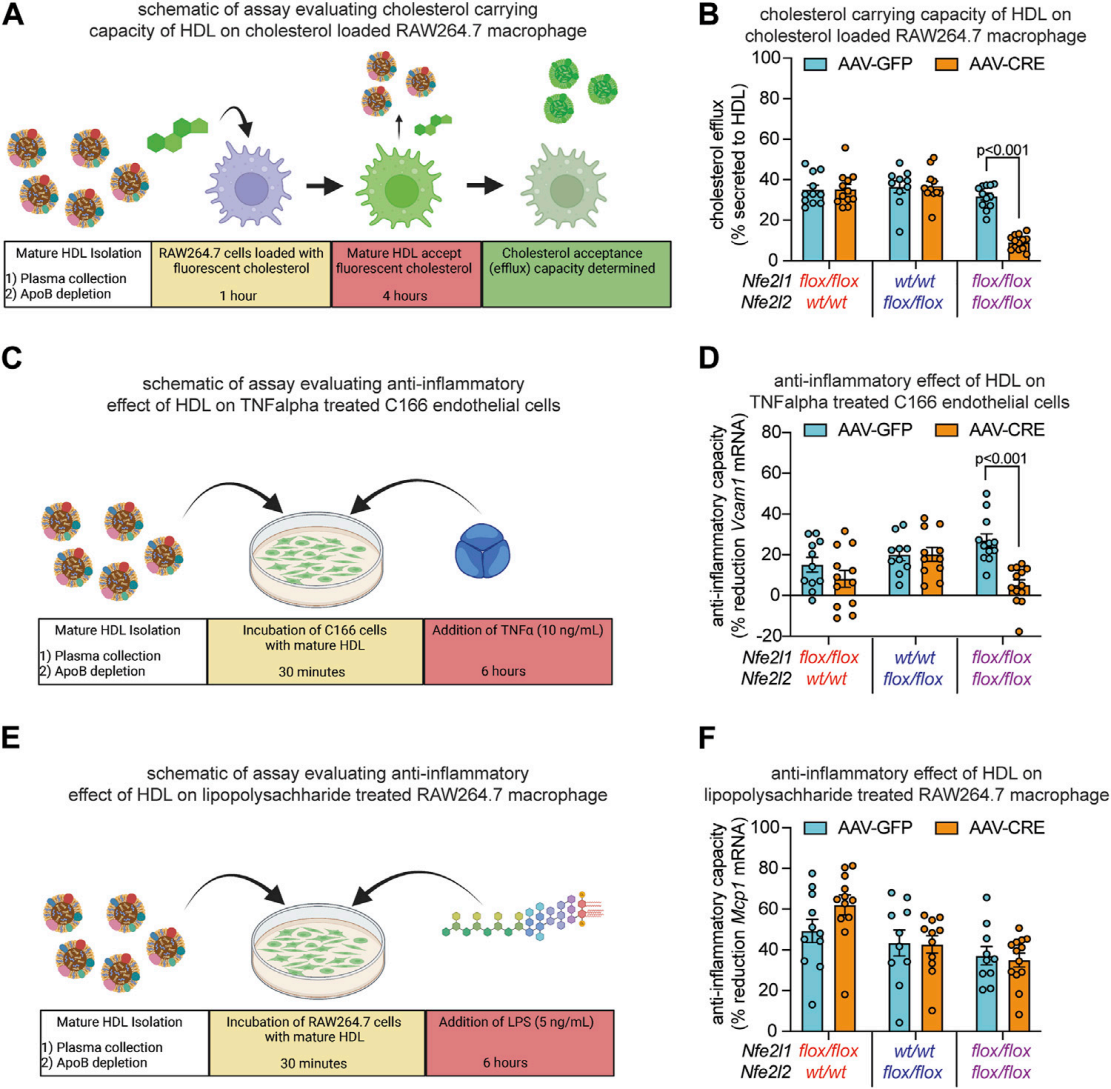
Combined hepatocyte deficiency of Nrf1 and Nrf2 impairs HDL functionality. **(A–F)** Study designs **(A, C, E)** and results **(B, D, F)** of HDL capacity to accept and carry cholesterol that has undergone efflux from RAW264.7 macrophage **(A, B)**, anti-inflammatory capacity to suppress TNFα-induced *Vcam1* mRNA expression in C166 endothelial cells **(C, D)**, and anti-inflammatory capacity to suppress LPS-induced *Mcp1* mRNA expression in RAW264.7 macrophage **(E, F)**, comparing mice with deficiency for Nrf1, Nrf2, or both (AAV-CRE) to respective control (AAV-GFP) mice (n = 5-6 males pooled with 5-7 females). The *p*-value was determined by t-test, adjusted for multiple comparison. Data in **(B, D, F)** are mean ± standard error of the mean, with points showing the value of each biological replicate. **(A, C, E)** were created with Biorender.com.

Since one of the most established functions of HDL is to mediate reverse cholesterol transport, we assessed this function by measuring HDL capacity to accept cholesterol from cholesterol loaded RAW264.7 macrophage cells through cholesterol efflux, according to schematic in Figure 4A. Relative to control, HDL from mice with Nrf1 deficiency and mice with Nrf2 deficiency had equivalent cholesterol acceptance capacity, whereas HDL from mice with combined deficiency had markedly reduced acceptance capacity (Figure 4B). Another function of HDL is to reduce inflammatory signaling. To evaluate, we assessed HDL capacity to counteract TNFα-induced *Vcam1* mRNA expression by C166 endothelial cells, according to schematic in Figure 4C. Relative to control, HDL from mice with Nrf1 deficiency or Nrf2 deficiency had similar anti-inflammatory effect, whereas HDL from mice with Nrf1 and Nrf2 combined deficiency had reduced anti-inflammatory effect (Figure 4D). As a complement, we assessed HDL capacity to counteract LPS-induced *Mcp1* mRNA expression by RAW264.7 macrophages, according to schematic in Figure 4E. In this case interestingly, the anti-inflammatory capacity of HDL from mice with Nrf1 deficiency, Nrf2 deficiency, or combined deficiency was not different than control (Figure 4F). These findings show complementary actions by hepatocyte Nrf1 and Nrf2 can promote production of HDL, that is, effective for transporting cholesterol and suppressing TNFα-mediated actions on endothelium, but these actions do not influence other anti-inflammatory effects of HDL such as that triggered by LPS on macrophage.

## Discussion

There is growing interest in the homeostatic regulation and pleiotropic functions of HDL and its relationship to health and disease as well as in understanding how HDL acquires atherosclerosis-promoting dysfunctional and proinflammatory properties (Navab et al., 2011; DiDonato et al., 2013; Riwanto and Landmesser, 2013; Dullaart et al., 2014; Huang et al., 2014; Rohatgi et al., 2014; Annema et al., 2016; Rodriguez et al., 2019). Hepatocyte stress resulting from liver injury or inflammation can alter HDL composition and function via acute phase responses, and chronic engagement of this program may underlie HDL dysfunction (Van et al., 1995; Gabay and Kushner, 1999; McGillicuddy et al., 2009; Navab et al., 2011; Rosenson et al., 2016; Laudanski, 2021; Webb, 2021). The contribution of hepatocyte stress defense signaling to the homeostatic regulation of HDL is unclear. In this report, we investigate the role of stress defense-promoting transcription factors Nrf1 and Nrf2 in mice fed a mild metabolically stressful diet. Our main results are as follows. First, combined deficiency of hepatocyte Nrf1 and Nrf2 reduces the level of circulating ApoA1 and HDL cholesterol, and this coincides with reduced HDL capacity to accept cholesterol efflux from macrophages and counteract inflammatory effects of TNFα on endothelial cells, whereas these outcomes did not occur in mice with deficiency for hepatocyte Nrf1 or Nrf2. Second, combined deficiency alters the circulating HDL proteome but, in this case, there is partial overlapping effect that occurs in single gene deficient mice. Third, alterations to the abundance and composition of the HDL proteome in mice with combined deficiency positively correlate with altered liver gene expression of these same proteins. Altogether, complementary actions by hepatocyte Nrf1 and Nrf2 appear to play an important role in promoting liver production of functionally viable HDL.

HDL protein composition is known as a key factor influencing its functionality (Gordon et al., 2011; Shah et al., 2013; Furtado et al., 2018), but there is incomplete understanding regarding how it is physiologically established and regulated, and the role of its impairment in disease. Hepatocytes produce and secrete the majority of HDL resident proteins into circulation (Timmins et al., 2005; Brunham et al., 2006; Shah et al., 2013; Furtado et al., 2018). Moreover, the liver is a critical mediator of stress tolerance and this process involves alterations to HDL (Shah et al., 2013; Strnad et al., 2017; Kubes and Jenne, 2018; Webb, 2021). Hence, coupling of liver stress to HDL composition and function may be a systemic stress-adaptive response and hepatocyte stress defense programs may control the magnitude of this response and its resolution. Here, we explore this relationship by deleting stress defense factors Nrf1, Nrf2, or both in hepatocytes of mice fed a mild metabolic stressor and then comparing the composition and functionality of circulating HDL to respective controls. Based on previous knowledge (Degoma and Rader, 2011; Gordon et al., 2011; Fisher et al., 2012; Shah et al., 2013; Zannis et al., 2015; Geller et al., 2018), reduced ApoA1 in combined deficiency likely caused the reduction in circulating HDL cholesterol as well as the reduced cholesterol acceptance capacity in functional assays. Reduced ApoA1 can also contribute to the reduced anti-inflammatory effect of HDL on TNFα treated endothelial cells, although there were also changes to other inflammatory molecules as well as clotting factors and the acute phase response-associated and iron transport molecule haptoglobin. Hence, stress-defense programming by hepatocyte Nrf1 and Nrf2 that influence HDL function may underlie multiple types of stress-adaptations to promote homeostasis.

In addition to qualitative features of the HDL proteome in rendering its function, the quantity of HDL particles is also critical. Thus, an important consideration of our study is that reduced circulating plasma ApoA1 level in combined deficiency is also an indication of reduced HDL particle concentration. To account for potential differences in HDL particle number, proteomic analysis of fractions 8 and 9 was normalized by protein content. With this normalization there was no difference in ApoA1 in fraction 9, while there were differences for several other HDL resident proteins. Thus, differences in HDL functionality in mice with combined deficiency likely resulted from altered secretion of ApoA1 and other HDL-resident proteins that in turn resulted in reduced HDL abundance and altered composition.

A still emerging concept is that circulating HDL has functions beyond reverse cholesterol transport (Gordon et al., 2011), and clinical evidence show these other functions may impact disease outcomes (Dullaart et al., 2014). The current study employed only a mild metabolic stress condition and thus had limited scope. That being said though, our primary intent was to investigate the physiological relationship between hepatocyte stress signal networks and systemic HDL function. Given our results, it would be interesting to examine this relationship in atherosclerosis-prone disease models as well as other pathologies in which liver stress tolerance plays an important role. Notably, tolerance to sepsis-promoting bacteria could be affected by complementary actions of hepatocyte Nrf1 and Nrf2, as this has been strongly linked to HDL functionality and the hepatic acute phase response (Carpentier and Scruel, 2002; Haas and Mooradian, 2010; Strnad et al., 2017; Kubes and Jenne, 2018; Laudanski, 2021). Moreover, related pathways in liver were the most profoundly affected in our previous study (Akl et al., 2023). Additionally, since Nrf1 and Nrf2 are known to promote oxidative stress defense in cells (Xu et al., 2005; Sugimoto et al., 2010; Hirotsu et al., 2012; Zhang and Xiang, 2016; Widenmaier et al., 2017; Yamamoto et al., 2018; Cuadrado et al., 2019; Mohs et al., 2021; Akl et al., 2023), these actions may also be linked to anti-oxidant functions of HDL that counteract systemic oxidative stress and tissue damage (Van et al., 1995; Undurti et al., 2009; Navab et al., 2011; Annema et al., 2016; Rosenson et al., 2016; Zhang et al., 2022). Future studies employing relevant experimental models will provide more clarify to this potential physiological relationship.

Overall, our study shows that hepatocyte Nrf1 and Nrf2 contribute to HDL functionality. The mechanism may involve stress-induced alterations to retinoid X-receptor transcriptional programming, as we found this to be severely disrupted in our previous work (Akl et al., 2023) and such programs are known to regulate liver expression of HDL-resident molecules (Kalaany and Mangelsdorf, 2006; Kersten and Stienstra, 2017; Kim et al., 2019). Future work focused on developing insight is warranted, as deciphering this mechanism may reveal strategies for manipulating HDL functionality to ameliorate disease. Interestingly, HDL from mice with combined deficiency had reduced anti-inflammatory effect on TNFα treated endothelial cells but not on LPS treated macrophage. Understanding differences and similarities in HDL particles that coincide with this discrepant outcome may be informative. On final note, this study inspires an interesting question as to whether enhancing liver stress defenses may affect HDL functionality via enhancing Nrf1 or Nrf2 actions in liver. The answer may open a promising pathway for effective HDL targeting therapy.

**Data and Code Availability.** All data are contained within manuscript. Raw mass spectrometry pertaining to proteomics have been deposited at the PRoteomics IDEntifications (PRIDE) Database repository (ProteomeXchange: PXD042103). All other unprocessed data corresponding to figures have been deposited in the Mendeley data repository (DOI: 10.17632/5mzvrgrhjn.1). These datasets will be publicly available as of date of publication. Accession numbers will be provided in Materials and Methods section. Any additional information required to reanalyze the data reported in this paper will be made available from the lead contact upon request.

## Data Availability

The original contributions presented in the study are publicly available. This data can be found here: This data has been deposited at the PRoteomics IDEntifications (PRIDE) Database repository (ProteomeXchange: PXD042103). All other data corresponding to figures have been deposited in the Mendeley data repository (DOI: 10.17632/5mzvrgrhjn.1).

## Abbreviations

AAV-CRE, adeno-associated virus CRE recombinase; AAV-GFP, adeno-associated virus green fluorescent protein; C3, complement factor 3; C9, complement factor 9; CAD, coronary artery disease; FPLC, fast-protein liquid chromatography; HFFC, high fat, fructose, and cholesterol; LPS, lipopolysaccharide; mRNA, messenger ribonucleic acid; Mcp1, monocyte chemoattractant protein 1; Nrf1, nuclear factor erythroid-2-related factor 1; Nrf2, nuclear factor erythroid-2-related factor 2; TNFα, tumor necrosis factor alpha; Vcam1, vascular cellular adhesion molecule 1.
