# Supplementary material for: HDL functionality is dependent on hepatocyte stress defense factors Nrf1 and Nrf2
Source: Front Physiol. 2023 Jul 12;14:1212785. doi: 10.3389/fphys.2023.1212785 (PMC10369849; doi:10.3389/fphys.2023.1212785)
Supplement: Supplementary file 1 [file Image1.pdf]

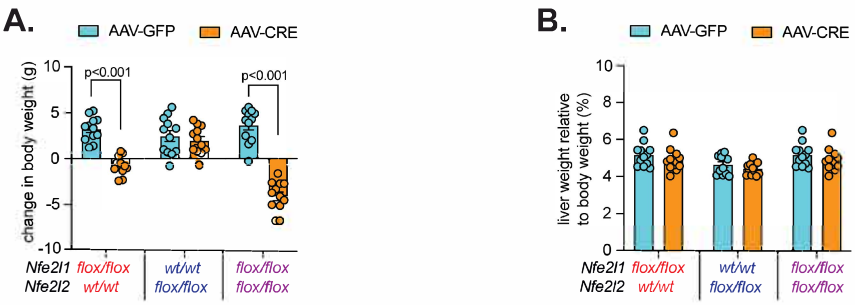

Supplemental Figure S1. Validation of deletion model. Mice were treated as shown in Figure 1A, with body weight measured at beginning and start and end point as well as liver weight following euthanasia. (A and B) Change in body weight, relative to initial day of infection (A) and % liver weight (B) for mice (n = 5-6 males pooled with 5-7 females). p-value determined by t-test, adjusted for multiple comparisons. Data in A and B are mean +/- standard error of the mean, with points showing the value of each biological replicate.
